# Complete Genome Sequences of Citrobacter braakii Strains GTA-CB01 and GTA-CB04, Isolated from Ground Beef

**DOI:** 10.1128/genomeA.01307-14

**Published:** 2015-01-08

**Authors:** Prabh Basra, Adam Koziol, Alex Wong, Catherine D. Carrillo

**Affiliations:** a Department of Biology, Carleton University, Ottawa, Canada; b Canadian Food Inspection Agency, Government of Canada, Ottawa, Canada

## Abstract

Citrobacter braakii is a Gram-negative bacterium belonging to the *Enterobacteriaceae* family. Here, we report 5.2- and 5.0-Mb genome assemblies for C. braakii strains GTA-CB01 and GTA-CB04, respectively.

## GENOME ANNOUNCEMENT

Citrobacter braakii is commonly found in water, soil, food, and the intestinal tracts of animals and humans (1). C. braakii has been associated with infections such as hospital-acquired bacteremias and urinary tract infections, making it an opportunistically pathogenic species (2). Citrobacter freundii, a close relative of C. braakii, contains *qnrB*-like genes that confer plasmid-mediated quinolone resistance to these species (3). Citrobacter is also of potential industrial interest, since some strains produce the animal feed supplement phytase. Microbial sources of phytase are promising for production of this supplement at a commercial level (4, 5).

C. braakii strains GTA-CB01 and GTA-CB04 were both recovered from separate raw ground beef samples (2013) in Ontario. Genome sequencing for both strains was performed using the Illumina MiSeq platform (250-bp, paired-end reads). Average insert sizes of 288 bp for GTA-CB01 and 304 bp for GTA-CB04 were obtained, and *de novo* assembly was carried out using Velvet version 1.2.10 (6). Annotation of the resulting contigs was carried out using the NCBI Prokaryotic Genome Automated Annotation Pipeline (PGAAP) (7).

The total assembled genome sequence of GTA-CB01 is 5,234,166 bp, assembled into 64 contigs with an average size of 83,082 bp and 32× coverage. PGAAP predicted 4,954 genes with 4,804 coding regions, 22 rRNAs, 85 tRNAs, and 7 noncoding RNAs. GTA-CB04 has an assembled genome sequence of 5,036,963 bp, assembled into 17 contigs with an average size of 296,291 bp and 52× coverage. It consists of 4,698 genes with 4,565 coding regions, 23 rRNAs, 23 tRNAs, and 1 noncoding RNA.

Various virulence and defense genes associated with adhesion and efflux (e.g., MdtB, MdtC, and MdtD) were found in both assemblies, which may result in pathogenicity in certain environments. However, *qnrB*-like genes resulting in quinolone resistance were not found. We performed an initial BLAST search for phytase production genes using C. braakii YH-15 phytase (accession no. AY471611), and found a strong hit at 98% sequence similarity between the gene and both of our assemblies (8). One contig in the GTA-CB01 assembly shows 96% sequence similarity to a 16,532-bp region of the pCRY plasmid encoding a type IV secretory system in the human-avirulent Yersinia pestis biovar Microtus strain 91001 (9).

These data suggest that C. braakii GTA-CB01 and GTA-CB04 are both nonpathogenic and produce phytase. Sequencing of additional isolates will help determine the frequency of potentially pathogenic strains and phytase producers.

### Nucleotide sequence accession numbers.

These whole-genome shotgun projects have been deposited in DDBJ/ENA/GenBank under the accession numbers JRHK00000000 and JRHL00000000. The versions described in this paper are the versions JRHK01000000 and JRHL01000000.
